# We are all angels: acting, reclaiming and moving beyond survivorship

**DOI:** 10.1007/s12682-014-0184-3

**Published:** 2014-10-09

**Authors:** Ariane B. Anderson

**Affiliations:** University of South Florida, Tampa, USA

**Keywords:** Survivor, Breast cancer, Illness narratives, Feminism

## Abstract

This article aspires to an embodiment of dynamic living versus mere survival. The term cancer survivor, including a survivor who is in remission, has been legitimated (Berger and Luckmann, The social construction of reality, p. 94 1967) by language which creates knowledge of what a cancer survivor is and does. Because we act under descriptions (Hacking, The social construction of what?, p. 103 1999), those of us who have passed through illnesses such as cancer not only have been given the name and the idea of survivor, we have assumed and conform to some or most of the characteristics assigned to it; examples of some of those characteristics are discussed throughout this project. Whether or not we choose to enact all that falls under the grammar of the classification of survivor, we still live with, create, and experience ourselves and others as legitimated by such a classification. The term survivor operates through a number of institutions (medical, capitalism, and media) resulting in individuals’ awareness of such classifications about themselves and others. Many, if not most, who are aware of being classified as survivors may wish to modify or resist the constraining aspects of those classifications and their descriptions. Through layered accounts of interviews and prose, I interact with this term as one who is both caught in and wants to go against the stream of classification and description. I want to transcend what I know, yet I am aware that whatever story I make and tell is a part of the whole—my story is part of two other survivor’s stories which I include in the following telling of my own. All of our stories matter. Still, I want to look beyond what is in front of me, move beyond it, dream. I do so with a desire to tell my story as part of other survivors’ stories.

## Introduction

Why does illness call for stories [3]? If we find ourselves with others in a medical setting such as a waiting room, we will hear someone’s story—whether we want to or not. When we do want to hear others’ stories, it is often because their stories act as maps that connect to our own stories. Despite knowing that chronic illness, disease, suffering, and death are all a part of living, when we experience them in the immediacy of our own space and time, we are surprised and compelled to make sense of them. We do this through stories, and as an extension, through existential questioning.

Here, through narrative inquiry, I examine the current culturally given survivor in remission identity/body, take it apart, and attempt to derive meaning from those parts (literal and figurative) as I recreate my remission identity in concert with two other voices. Frank [4] says that “ill people… learn by hearing themselves tell their stories, absorbing others’ reactions, and experiencing their stories being shared.” I agree that we begin to make sense of our experiences through the shared narratives we conceptualize and reinforce and transmit between lives. I sought to accomplish this kind of learning when I conducted interviews with my friends and fellow breast cancer travelers. Our interview experiences provided a unique meta framework wherein we could unearth aspects of our stories that we might otherwise skip over in our interpersonal exchanges together, we create what it means to be a survivor and beyond.

## The image of survivor

Formerly portrayed as disempowered, stigmatized, disfigured, and certain to die [5], the current social configurations and cultural symbols of the breast cancer patient extol women as survivors, heroines who are “triumphant, happy, healthy, and feminine” [6–8]. When attending a breast cancer survivor race or walk, the language used rarely suggests that the breast cancer sufferer is a victim. Instead, she is an inspiration to all who see her: she is the embodiment of an empowered member of the sisterhood of survivors. Not only is she victorious (even if she is in stage IV cancer and unable to walk) she also exemplifies the transformative ability of the disease experience as a way of becoming a better human being. Despite a grammar of empowerment, contemporary definitions of the breast cancer survivor identity, while commodified as pro-woman, are not necessarily pro-feminism [9, 10]. Instead of resisting dominant definitions through a questioning of the structural causes and perpetuation of the disease, as well as questioning social inequalities, the breast cancer survivor and her supporters through the enactment of a free market feminist [11] volunteer and buy for the cause and are impelled by directives, i.e., “Race for the Cure,” issued by entities such as the Susan B. Komen Foundation. The breast cancer survivor often is obscured by or rendered invisible by such meanings. What if she does not fit the survivor description? What can she do to enact compassion and awareness while dwelling in the dialectic of disease sufferer and survivor? What if she does not feel victorious? Is she truly a “survivor”?

But is there a universal definition of cancer survivorship? Since the term “cancer survivor” was created almost 30 years ago, its broad interpretation and usage have been generally undisputed, defining virtually anyone from the moment of diagnosis, a survivor, [12]. Researchers who study the use of the term claim that the sheer diversity of populations afflicted with cancer, who have moved beyond treatment, is reason enough to question the universality of the term. Moreover, some cancer victims find themselves conflicted about self-identifying as “survivors” because they do not relate to the concept of beating the disease [13, 14]. Although recent nursing oncology literature suggests that positive life changes and posttraumatic growth (PTG) can result from intensely stressful events such as breast cancer [15–17], in contrast, studies of Arabic, Norwegian, and Chinese women’s experiences of chemotherapy found negative psychological, social, physical and familial effects [18]. In spite of cultural messages of positive survivorship identity, some women choose to construct different meanings of, “survivorship or reject the term survivorship” altogether [19]. Women like me, well—we define ourselves as anything but heroes.

## Looking for light

This is my story right now, a de jour crafting of identity fragments and composites, cobbled with the meanings I perceive and impute together with my social world and my faith in the spiritual. I suspect that we all have moments in which we realize that our interactions with others seem to be driving our sense of self, an almost out-of-body salient sense of self. I have daily crises, sometimes moment-to-moment crisis of identity which necessitate that I revisit and re-story myself through interactions with others. Here, I have invited my friends along to help me compose today’s chapter. Together, we do this within relationships where we swap accepted stories about what our identities should look like based on the situation or group in which we find ourselves. As three bodies in remission, each of us in some fashion identifies as a survivor, but each also, in our own way, resists the language of disease that attempts to contain us. Today’s truth for me will be shaped by stories that are presently recalled as a member in a community of survivors. Such is the case of one of my identities, one that I embrace awkwardly—the breast cancer survivor identity narrative.

Since being diagnosed with breast cancer 4 years ago, I have written in the midst of, not necessarily through, crisis and have seen the value of narrative therapy [20, 21]. I attempted to face down my fear of death through writing personal prose, poetry, and scripts about the fear of the unknown. I did this to counter the absurdity and containment of the languages of cancer; for example, the institutional pronouncements:

“Patients with Stage 3b-4 diagnosis may survive for up to 2-5 years”

“Uninsured and underemployed? You still don’t qualify for Medicaid or indigent healthcare”

“You must pay for the mammogram in full before we render service.”

“Ma’am, the Cancer Society does *research*, we don’t fund patient care.”

Or people’s utterly unhelpful sentiments:

“God doesn’t give you more than you can handle.”

“You know, sadly, my wife who was a Chemistry teacher, lost her memory because of ‘chemo brain’. Let’s hope it doesn’t happen to you!”

“At least you can get a new set of bigger and better breasts! The ones you’ve always wanted, right?”

But here is what I really thought about my cancer diagnosis:

In the curve of afternoon sun

I wonder if

Darkness will not be as dark as they say.

I wonder if endings will be traced with gilded prisms

Full clouds hung down with palpable groans, terror, velvet black

unblinking, enveloping feeble frames,

yet replete with linings of splendorous *eldil.

In this sealed blind underworld,

no fissure of luminosity here,

can dark be light?

Dark rays jar loose fallow dreams

My head rattles. The dying matter, no matter

Such things square off at high noon, cancelling each other

This day, this night

light is a prize fought for

What was Shakespeare reading on his deathbed?

Did he hold onto those words for dear life?

I’d be content with a sliver of light in my palm.

*cosmic angels in C.S. Lewis’ *Space Trilogy*

## We catch them

“When I have been blessed to recover, I feel like I’m here for a reason: to get other people through this.”

Anne Creech, breast cancer survivor

Connie, an 8-year survivor, is well known in the community for chairing fundraising initiatives, walks, events, as well as working for a breast cancer support business. She sees her identity in what she does through her body and how cancer affects other women’s bodies. So attuned is she to what other women may be feeling, when she tells a story about her experiences with the female clients that she works with, she does so with the precision of a reporter. Indeed, Connie embodies, her identity is founded in idea of support. She is an example of courage and steadfast determination as well as compassion and support.

I would not be here doing this job, had I not had breast cancer. Some women come in and some of them have come directly from the doctor’s office and just hearing the words, ‘You have breast cancer,’ and sometimes they walk in the door and you can see through the body language that something’s really amiss and they make it to the counter and put down their prescription and their mouth opens, and a big sigh comes and they say, ‘I have…’ and the tears flow and the body slumps… and we hope to have arms there to say, ‘honey, it’s okay.’ The women understand because we’ve been there. They need the hug, the arm to catch them, because the body just slumps, because they finally realize that ‘Well, maybe I’m someplace safe; maybe I’m somewhere where this is going to *make sense.*

And while it’s nice to have beautiful breasts and it’s nice to have a symmetrical figure, what I have seen is that women who have been through breast cancer want validation that they have been through something so life-changing and so important, that they want someone to say, “Honey, I understand” because it is life-changing. A lot of women come in here grieving because they lost a body part or maybe they’ve had breast cancer and have lost their husband and since I have my own scars, I know they just need that smile and that comfort from another woman who knows that they have been through the fire.’

Connie

## Being able to smile and mean it

I pull my late model car into the parking lot of the Lake County YMCA. I am here to meet my friend, Marsha. It seems frantically busy as luxury SUVs and sedans zip back and forth past the front entrance. I finally reach her and we grasp each other in our typical fierce embrace. Her wide smile reveals beautiful white teeth which highlight her green eyes and strong, tan face. She has a short light brown streaked bob hair style. (Is that a new wig? I wonder how she teaches yoga without her wigs falling off.) We turn and enter through the door to the front lobby and are met with the mass of loud activity of exercise machines, group fitness classes and crush of people. We make our way back to an office space shared by several people, take a seat at a small lunch table and with the din behind us we begin the purpose of my visit. Marsha has consented to an interview with me and this needs to happen immediately because she has to teach a class in 35 min and she is one busy person. We get settled and I go over the interview protocol with her. As I explain the procedure, I remind myself that as we interact, some things might be triggered; I must remain open to a range of feelings, surprises, and perhaps discomfort. As fellow cancer survivors, I sense that we together, are entering a new phase of storytelling where the dynamic of meaning making has the potential to affirm or disrupt our ideas about our identities as women, our bodies, and our futures.

### I press play and the interview begins

Marsha, thanks for consenting to this interview. Tell me a little bit about your professional life.’ She sighs, ‘well, my career has been unbelievable; I’ve owned four businesses, been in the corporate world, top of the heap: a human resources director, a sales director… Um, I’ve done it all. I’ve traveled all over the country hiring and training managers for these corporations, a fitness instructor where I teach Pilates and yoga. And then I became a speaker and I’m a writer—I’m a Dale Carnegie training speaker, I write greeting cards for a company in New York. I’ve written for Detroit news editors & several magazines, whatever it is that they wanted—you know, how to open a business, how to run a franchise, how to have a baby, how to apply makeup—whatever people were dealing with that time—that platform is what I wrote on or spoke on. I sell insurance…

She goes on:

My goal my whole life, Ariane, is to learn one new thing every year. One year of learning how to drive a stick-shift; one year it was to become an accountant; whatever, I just have a goal every year I’m going to learn something new. Pretty much I have, you know, and some years no, because of illness. I’m a make-up artist. I had to go to Los Angeles and learn that when I had my cosmetic studios—I had two of them—so I could do make-up for fashion shows and beauty pageants and show women what colors to wear and how they should put it on. And I had a flower shop, and so I won awards for my floral designs. I was on the Board of Directors for one of the major floral wire service companies. I had a restaurant…

Wow, I think. I had no idea she has done all of this. And then I think, ‘Is this all for real? How can one person do all of this? This sounds too good to be true, like Frank Abagnale Jr. in “Catch Me If You Can.”’ We had not even gotten to what I knew about her illnesses—she had one of the most traumatic cancer stories I had ever heard. I was not sure what to think about what she had told me so far, except to believe her.

Here I am (Ariane), 4 years in remission and a “survivor.” Despite my herculean efforts at non-conformity, I am part of the cadre of archetypal survivors who are positively weighed down with pink sequins, boas, t-shirts, stickers, crowns, buttons, banners, balloons, halos (yes, real halos) and every conceivable marketing insignia—all in effect broadcasting a status of disease warrior-princess.

But I am depressed. ‘Why are you depressed?’ you ask. ‘You’re alive. Be grateful, there are so many people who die from cancer. You’re fortunate to have the technologies and therapies that you have had.’ ‘Why aren’t you cheering newly diagnosed cancer patients on with the same encouragement that you got?’

Well, I have done all that. I have done the walks, the runs, the fundraisers, the parades, the talks with other women scared out of their minds, the talks to other women because I am scared out of my mind, the volunteering, the writing, the research, the studies, the fashion shows, the calendar shoots, the PINK EVERYTHING. I have pink fatigue [22]—more than you can imagine. I am not depressed about all of that, in fact, I am happy to be part of those communities. Together, we build each other up. However, the critic in me thinks there is plenty to be depressed about when considering that despite an annual billion dollar global philanthropy industry, there are still so many people who are getting sick and dying from cancer and it seems like few people (least of all the sufferers themselves) who dare to question who is profiting from it. In hindsight, I thought my illness experience was enough to cure my depression, to cure the persistent thought that I am inherently defective, to wipe all the wreckage away and start over with a clean slate. But grief has a curious effect on the body and it has resurrected my nemesis.

I have served for five years on *Making Strides Against Breast Cancer*, and through the local *Angels of Breast Cancer* calendar projects tens of thousands of dollars have been raised and given directly to the *American Cancer Society*. I am not sitting on the executive committee this year, but I am a team captain of the store. That’s big, when you hear the effort has raised over 155,000 dollars, that’s pretty rewarding. From time to time the *ACS* calls on me to be a cancer survivor speaker. It’s been a hard trip surviving, but there’s been a lot of rewarding experiences, and I would not be here doing this job, had I not had breast cancer. I would have been sympathetic to the cause, but I would not have known the level of experience and the commitment it takes. Having been diagnosed with stage 3C cancer and undergoing a radical bi-lateral mastectomy and surviving for eight years, I believe that God gave me extra days and He expects me to do something with them. That’s why I do the work that I do.

Connie

### I nod my head

‘It sounds like you are a business-woman extraordinaire, Marsha!’

### She smiles big and says

Yes, I learned business as a baby, literally at my parent’s feet, they took me to work with them every day (which is something I did with all my four sons as well). I learned how to interact with others by listening to them. It’s always been a gift. I learned way back that selling myself is number one. I’ve always had a penchant for helping people get started and get in the business world and start businesses—I’m an accountant as well—and show them how to keep their books. So it’s been fun. *God has gifted me with many experiences so that when something comes up I’m able to help so many people and I think that’s what my life is all about.*

At this point, I look down at my notes. My eyes blink, and I feel my throat tighten as I think about my own lack of motivation, organization, and achievements in comparison to Marsha. I wonder if she ever has experienced any chronic depression and anxiety—the kind that has reclaimed my life—the kind which on occasion incapacitates me as a student, instructor, caregiver, and a supposedly mature adult. I keep looking down pretending to read, overwhelmed by feelings of inferiority, shame coursing through my upper body. I feel a disjuncture at this point, a divide from Marsha that threatens to obstruct the meaning that we have shared for the past several years. If I brought up the term depression, would that kind of vocabulary “facilitate or obstruct” [23] our interaction? Would Marsha think less of me? I suspect that despite the cultural saturation and so-called acceptance of our current societal idea of depression (for example, the ease with which pharmaceutical advertisements talk about it), it still remains an undesirable state in which to find oneself. I know from my own personal experience that even though I experience depression, I avoid the discomfort that a depressed person exposes me to as if it is a communicable disease. I also know the accompanying feelings of helplessness when I know that I cannot ‘fix’ another’s melancholy or worse, a mental illness ‘diagnosis.’ At this point, I am not confident that Marsha knows how it feels to fight this particular kind of battle.

It is true that her own illness story is one of epic proportions: she had been on her death bed three times, had permanently broken ribs, a radical bi-lateral mastectomy, no chance of reconstruction of her breasts, an incomplete head of hair and she had had a tumor removed from her heart. She taught Yoga and Pilates daily to deal with the chronic pain that she would live with the rest of her life. But here she sat, without any indication that she was in physical or mental pain. In fact, hope and love were radiating out from her. I could feel it, but not for myself. How and why did I manage to overcome a serious cancer diagnosis and treatment only to end where I am now—feeling hopeless about myself and my accomplishments? This question hits me with force. I have failed to measure up to the pink warrior ‘sheroe’ narrative [14]. I have survived cancer only to wonder why I am alive. In contrast, Marsha seems to be the embodiment of… what? Does she measure up to the image of a cheerful, unquestioning, fiercely determined survivor? I am about to find out more. I look back up at her open, reflective eyes and say a bit faintly

You really have done it all!

The phone rings loudly in the space and the secretary’s conversation distracts us momentarily. Marsha winks at me and we continue.

Knock Knock

Who’s there?

Cancer.

Cancer who?

I have cancer

That’s not funny, that’s very serious

Well let’s make it funny

How am I supposed to make it funny?

Cancer Jazz-Hands

So, soon after my cancer diagnosis, I made it funny. I employed gallows humor at every turn, partially because that is my personality and because I thought that was the appropriate way to deal with it. I turned a death sentence into an excuse to perform bad-ass to everyone and everything. I occasionally told people who annoyed me, I have cancer, do not fuck with me. And you know what? It worked. It worked so well for me that I let go of decades of wreckage that I had allowed to hold me back. And while I was taming my depression demon, I met a man who let me be myself and together we made it through the valley of the shadow of death. I had a new attitude, it seemed, and someone to share with what time I had left.

I look in my mom’s checkbook and see that she is been signing checks, big checks, checks that my partner, Steve, unbeknownst to me has been getting her to sign. I wonder what made me pay attention to this all of a sudden, especially since I had turned a blind eye for so long.

An inner voice directs me to look.

For 4 years, I have trusted him implicitly.

I feel myself go cold with shock.

We were supposed to be together forever—I will never leave you nor forsake you.

When I lay on my bed, sick from chemo, despairing of life, he told me: You’re not going to die from this. Here’s how you’re going to die. I’ll be 94 and you’ll be 93 and I’ll be driving us someplace and the last sound we’ll hear is of you screaming as we go over the cliff that I accidently drove over. *That’s* how you’ll die.

That is what kept me going, head down, facing all of the invasions, breeches, violations of cancer treatment in my body and mind. In this place of particular vulnerability, I had fully trusted that I had finally found my soul mate, the one with whom I could unashamedly share the ‘real’ me. He had not run from me when he found out I had breast cancer, he stayed with me. His hands had gently kneaded out the tension from my shoulders, rubbed the anxiety from my forehead, steadied my faltering steps, carried my body in and out of treatments; he had smoothed and traced his fingers unhesitatingly, lovingly over the scars that used to be my breast. He had been my rock, unflinching in his acceptance, understanding, and adoration of me, of us. The rock dashed me to pieces. This was not supposed to happen. I had survived the monster only to find another one had appeared. My depression-free liminal space was foreclosed.

Okay, at this point I’ve had 22 surgeries, umm, I’ve always been pretty healthy, but 13 years ago this month, October 8th, I had the bilateral mastectomy’… Marsha goes on about her doctors finding a tumor the size of a golf ball in her heart of all places, how she died several times, got stage IV cancer again… and I just said that’s it. I’m all done and I’m not doing anymore, no more chemo, nothing, leave me alone. So I went home and I wouldn’t do anything. The doctor sent me for therapy, put me on anti-depressants, I became a vegetable practically, I couldn’t focus, I wouldn’t eat or drink or talk. I was severely depressed. Because I had just gotten over this horrible heart thing that one in a million people do and most of them don’t even live so, of course I should’ve been grateful…

As I listen to Marsha reveal her vulnerability, I relax. I begin to feel a tiny bit safer. Perhaps, she does know what it feels like to feel hopeless for long periods of time. I know what *that f*eels like.

I say,

It sounds like you were profoundly ill. What questions came to your mind? ‘I went through all this for this?’ ‘What does it all mean?’

She replies,

Oh yes, I could understand why I was sick the first time, so they could discover the tumor which would have killed me, but getting stage IV cancer after that? I don’t understand why it came back but I’m sure I will someday.

I thought about the not knowing and how that could cause despair and I realized that Marsha faced those questions and responded like I would.

A final question, how do you define health? Just tell me in a couple sentences, what is health to you?

She did not hesitate,

It’s being able to smile and mean it.

And maybe sharing little bits of this story when God tells me to. Like in class, I don’t typically. But occasionally He’ll say, “Tell somebody,” and I might say “Yoga’s brought me God, Family, friends, and Yoga has brought me through cancer twice.” And they go, whoa, if she can do that, I can do it too; if you can do it then I can do it. So that helps them. But I don’t want to, I don’t want to wear the pink ribbons and bows pointing out that I’m a cancer survivor. I want to be normal: More than a survivor.”

I add,

Yes. We’re more than survivors. We’ve got to come up with a term that’s more meaningful than that.

We’re conquerors. We’re all angels.

I call to mind that Parry says that “the realization that we are all characters in each other’s stories as well as our own reminds us that our stories only go forward as we act in ways that also forward the stories of others” [21]. I am having that realization as Marsha and I share her story. We are moving each other along and the dynamism in the room at the moment is palpable. It is almost a presence. I feel encouraged, validated, and comforted, all at once: this, in spite of not having any answers as to why chronic depression and cancer have afflicted me or Marsha; this, despite not knowing if my depression will ever go away. I suspect that I do not need to know, I just need to keep creating meaning with the stories that I hear and tell.

## We are all *angels*

Lately, I have been wondering what to do with this body that has resisted cancer enough to get healthy again—a body that has succumbed to depression—a body that wants to resist the extant texts and canonical discourses that keep telling me that I have to be happy and cheerful. I am told that I am ‘here for a reason’ as if my suffering has a noble purpose (and an AIDS patient does not?); I have ‘work to do’, with a body that will never be the same; I have ‘kicked cancer’s ass’ but cannot kick my own; I have gotten well, only to be… what? What exactly is remission? Is it a break? Is it like an intermission? Do I go back to the show after this? If I go back to it, do I get more of what I had before intermission? Bodies in remission dread a cancer redux. Those yearly checkups are an exercise in ambivalence: fear and hope tumble into confusion about what is or is not metastasis, flesh and machine, what the past and future should look like, what Eve Ksosfsky Sedgwick refers to as “supposed oppositions that structure an experience of the self” resulting in “decades of free-fall interpretive panic” [24]. And I have been in a yearly free-fall, dealing with repeated high marker numbers, strange lumps and pains, and physical conditions that have indeed affected my body and mind. But I do not want to leave it there. I know that Marsha and Connie and many other people who have stories similar to us go through the same kinds of passages. When we talk to each other, we can ground each other, at least for a while. Our stories have the potential of dynamically effecting a looping action that Hacking [25] refers to as an evolution that occurs through interactions. Simply having the knowledge about how we have been classified allows the potential for change for us to see ourselves differently, for widely held perceptions to change, and for the opening of new ways of experiencing ourselves.

Reclaiming my body, my voice, is what Arthur Frank [26] tells me is what I need to do. But something in me does not want to revisit the old me or even the present me. I do not want an identity makeover, I want to find, create a new identity out of the tapestry of my suffering: to be agentially free from the constraints of the discourses, texts, and ruling relations that render bodies and identities invisible, disciplined. I want agency over what is. It is *not* what it is; it is what others and I dream it to be. With the help of my fellow storytellers, I want to tell my story “with the inventive power of new language and fresh metaphors” [27] to produce a unique, corporately composed volume of hope that fearlessly questions, and is breathed, honed, and fleshed out as our language and stories develop and loop back on themselves.

It is still not clear what I desire or should desire. The body resists and cannot be contained by language, but is constructed by it. I/we must continue to look beyond current deficit discourses to find a language of abundance to construct a wounded body and identity. Whether I get sick or stay well, move in and out of depression or stay suspended in it, this body wants to be loved, loved on the outside, inside, and loved chiefly by me.
